# Hilliard Seigler, M.D. and the origins of kidney transplantation and immunology at Duke

**DOI:** 10.3389/frtra.2023.1196455

**Published:** 2023-05-22

**Authors:** Stuart J. Knechtle, Justin Barr

**Affiliations:** ^1^ Duke Transplant Center, Department of Surgery, Duke University School of Medicine, Durham, NC, United States; ^2^ Ajmera Transplant Center, University of Toronto, Toronto, ON, Canada

**Keywords:** kidney, transplantation, immunology, HLA, MHC typing, histocompatibility

## Abstract

The contributions of Dr. Hilliard Seigler to the founding of the Duke kidney transplantation program were considerable in both surgery and immunology. Some of these highlights are summarized based upon interviews with Dr. Seigler by the authors.

In 1962, Duke School of Medicine Dean Barnes Woodhall and Surgery Chairman Clarence Gardner recruited Dr. D. Bernard Amos as Professor of Experimental Surgery and Immunology with the intent of beginning organ transplantation at Duke on a scientific basis. Dr. Del Stickel was assigned the surgical task of developing kidney transplantation. In 1965, Dr. Hilliard Seigler completed his surgical residency at the University of North Carolina, Chapel Hill and contacted Dr. Amos to arrange a fellowship in immunogenetics as a means of pursuing a scientific platform for organ transplantation. Using Dr. Amos' background in tissue typing and serologic matching, the team decided it would be best to start with living donor kidney transplantation. Although some individuals were opposed to living donor transplantation on ethical grounds, the Duke team advocated that living donations based on tissue matching would be most successful and discussed living kidney donations with an *ad hoc* ethics committee at Duke, a precursor of the current system of institutional review board. While there were different opinions in the ethics committee, the final decision supported the notion of living donor kidney transplants with donor–recipient pairs matched according to objective immunologic testing methods.

**Figure 1:**
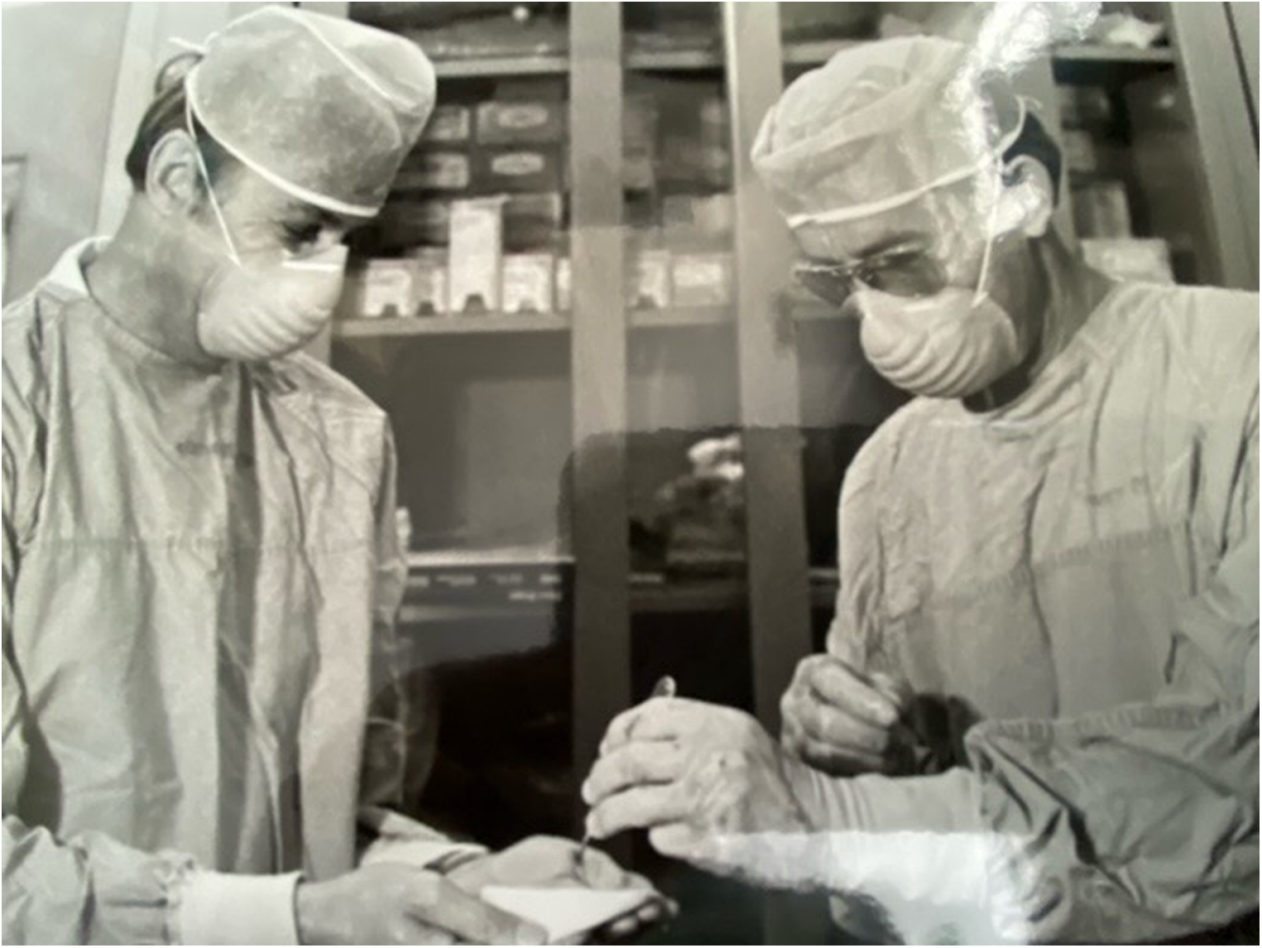
Dr. Hilliard Seigler on right performing skin grafts in laboratory.

**Figure 2:**
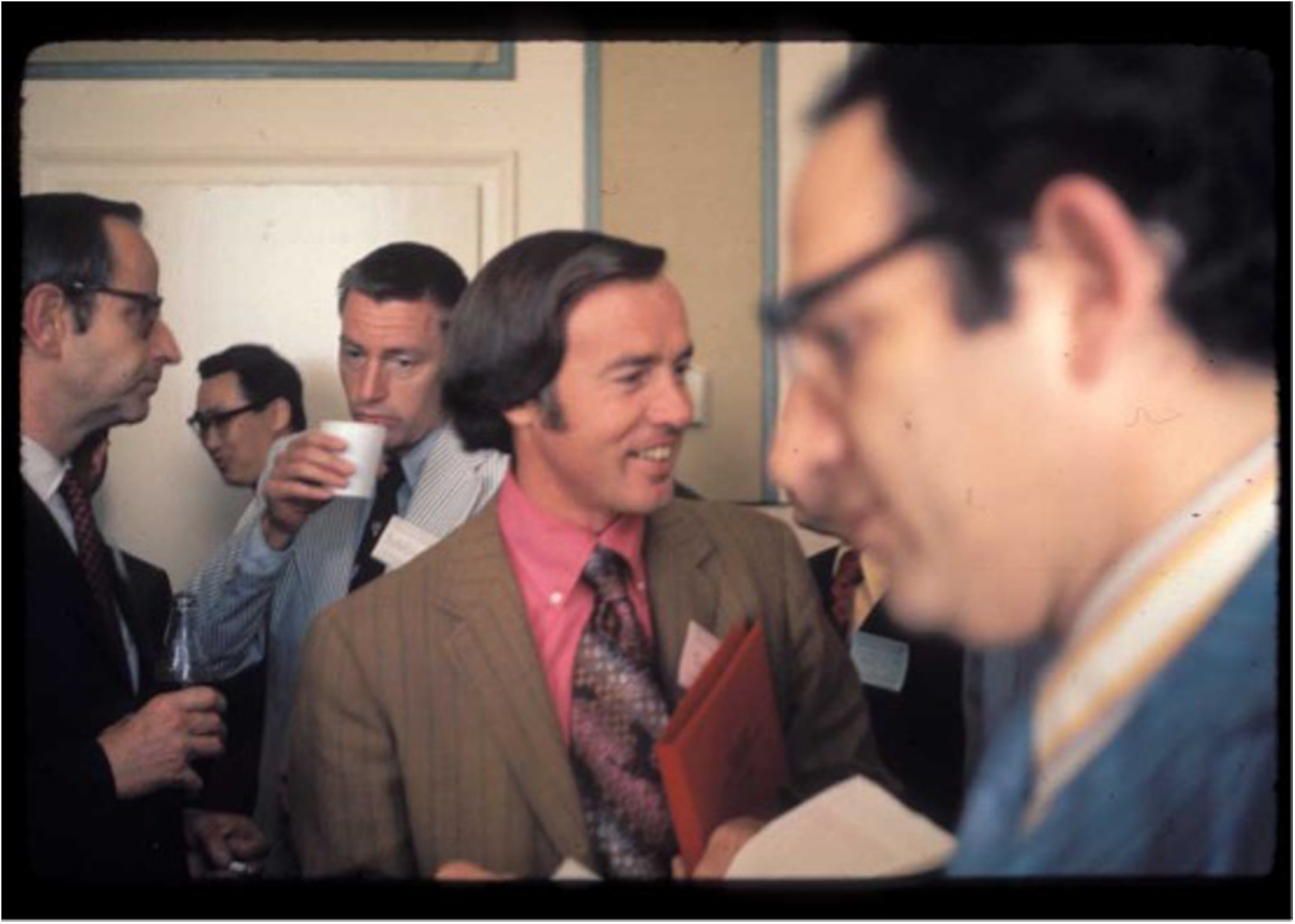
Dr. Hilliard Seigler, center, at a transplant meeting; Dr. Clyde Barker behind him.

Dr. Seigler was part of the group that trialed punch skin grafts as a screening tool for compatibility, placing skin grafts from prospective donors onto recipients and measuring responses (Figure 1). In addition, the investigators placed multiple skin grafts on each other in order to serve as controls and to validate methodologies. The serologic testing of recipients relied on approximately 30 panels of human lymphocytes to screen alloantibodies and became a particular focus of the group. They quickly learned that in order to make sense out of the genetics of a major histocompatibility complex (MHC), antigens that inbred patient populations would be needed as a starting point, as well as sharing such immunogenetic information across transplant groups. This prompted Dr. Amos to host the first international histocompatibility workshop at Duke in 1964 with the aim of sharing and standardizing assay methods and reagents. These workshops continued biannually until 1970. With respect to inbred patient populations, Seigler explained,

We got the most helpful information at Duke from two groups, even though we had looked at the Bantu tribe in Africa and an Indian tribe in Guatemala. We got the most information from tiny little towns in the mountains of North Carolina, which had been relatively closed for generations. They were little areas where there wasn’t a lot of travel to or from, and the families were quite large. We had 6, 8, or 10 children in the families, and we got incredible genetic information out of those.

Another group that we worked with was the Amish, and we worked with them in Lancaster, Pennsylvania. They had incredible Bibles that would have five to seven generations that were documented, so we got a lot of information in terms of understanding the genetics of these antigens that we were looking at. That was what eventually became known as the major histocompatibility complex in man or HLA.

The name HLA for the human MHC was assigned at the first histocompatibility workshop in Durham in 1963. Paul Terasaki proposed that the locus be named LA for Los Angeles (his location), while others felt that this was too specific to Dr. Terasaki. Bernard Amos, borrowing from the mouse MHC nomenclature (H2), proposed that merging H with LA would blend the letters and be a satisfactory compromise as “HLA.” Amos' motion was accepted. Although subsequently others have claimed that HLA stands for human leukocyte antigen, the above story is the actual history of the name.

The ethical controversy about proceeding with clinical kidney transplantation at Duke weighed on the first pioneers of transplantation at Duke, including Del Stickel, who performed the first transplant in 1965. He and Seigler proceeded despite the negative feelings of some members of the ethics committee. A professor in the law school alleged that “Del Stickel and I were immoral and non-ethical, because we were taking normal organs out of normal people and putting them in somebody else.”

The transplant program started only with living donors. In fact, the first 7 years of kidney transplants consisted exclusively of living donor kidney transplants based on thorough immunogenetic testing. Bernard Amos, recruited to Duke as an internationally recognized mouse immunogeneticist, also developed a reliable and consistent serologic screening method in humans to detect preformed antibodies, aiming to avoid early antibody-mediated rejection. Amos attracted multiple surgeons, such as Seigler, and other clinicians and scientists interested in learning transplant immunology. Seigler completed an NIH-supported fellowship in immunogenetics with Amos, identifying tissue antigens and determining what role they did or did not play in the human immune response, specifically organ rejection. His second goal was to work toward the elusive goal of transplant tolerance.

Based on the extensive typing of donors and recipients, including placing donor candidate skin grafts pre-kidney transplant on recipient candidates, the results of the living donor program led by Del Stickel, Bernard Amos, Hilliard Seigler, and Everett Anderson (urologist) proved to be excellent, relying initially on low-dose azathioprine (50 mg/day) without steroids as immunosuppression (1) in HLA-identical donor–recipient pairs. The 50-year follow-up on those early results was recently reported by Seigler et al. and compared to national outcomes, exceeding them by a wide margin (2) and supporting the validity and impact of pretransplant immunologic optimization of donor–recipient pairing. Seigler also investigated the relative influence of haplotype mismatches using skin grafting and mixed lymphocyte reactions (3).

In addition, Seigler played an instrumental role in the establishment of the Southeast Organ Procurement Foundation (SEOPF), the precursor of the United Network for Organ Sharing (UNOS). As Seigler tells the story, it involved Dr. David Hume of Medical College of Virginia:

Dave Hume had been at Medical College of Virginia, and he’d come down from Harvard. Bernard Amos and myself and Del Stickel drove up to Richmond and we talked to Hume because Hume was just turning out transplants but was having a lot of rejection. When you have a lot of rejection you get a big pool of patients that have rejected, and they’re very difficult to do a second, or third, transplant.

He was very sensitive to the fact of, “Look, I’m a force, but I need a way to get these difficult patients off of my list so we can go forward, and you guys down there at Duke are all about this typing business—maybe we ought to get together.” We established what we called “The Southeastern Organ Procurement Foundation” or SEOPF. We recruited other people, we got the University of Virginia involved, and the University of North Carolina was involved. We had as a great donor source the pathologist at Grady Hospital in Atlanta because they had a lot of potential donors, and so we got together as a group for organ sharing. SEOPF eventually became UNOS. That started here (Duke) also just like the histocompatibility did.

Another significant research activity of Seigler during those years was to collaborate with Dr. Paul Ebert, a cardiac surgeon at Duke at the time, in performing canine heart transplants and considering non-human primate heart transplants. Seigler and colleagues compared the immunogenetics of chimpanzees and humans, finding considerable similarities between the tissue antigens of chimpanzees and humans. Chimp and human cells were often totally cross-reactive with respect to eliciting identical immune responses in mixed lymphocyte reactions. At that time, chimpanzees were being considered as potential organ donors to humans, prompting the MHC typing of animals at Yerkes Primate Center in Atlanta as well as the colony at Rijwik in Holland. That work also extended into typing gorillas and orangutans for comparisons to humans. Dr. Keith Reemtsma, a transplant cardiothoracic surgeon in Louisiana, had actually performed approximately nine chimpanzee to human kidney transplants, several with a long survival of many months. Reemstma and Seigler communicated about this work because of the relevance of immune typing to the xenotransplant model. As Seigler explains, “He (Dr. Keith Reemtsma) sent me slides from a lady that he had transplanted chimp to human. This was a biopsy about 9 months out. The slides looked like it was an HLA identical transplant.” Eventually, however, a cellular immune response and severe [digoxin] toxicity occurred, so no long-term results developed because the patient succumbed to a cardiac arrest.

Seigler and his Duke colleague Paul Ebert had started canine heart transplants in the laboratory, and after achieving technical success they wanted to expand in 1967 into human heart transplants or xenotransplants, considering non-human primate donors based on the immunologic typing experience (4–7). However, this intention was not realized, although Seigler's background with non-human primate typing did prompt a clinical experiment of cross-circulation of a chimpanzee with a child in hepatorenal failure (8, 9).

Dr. Seigler's career pathway, while continuing involvement with transplantation, shifted to surgical oncology due to the clinical needs of the Duke Department of Surgery. He developed the melanoma clinic at Duke and led major innovations in clinical oncology research. He quickly became the most efficient surgeon in the Duke operating room, beginning his cases at 7 a.m. in a system where scheduled first starts began at 7:30 a.m. He was known for wheeling patients in and out of the operating room or even mopping the floor himself, charming assistants and staff with wit and enthusiasm. His mentoring in transplant immunology influenced the careers of Drs. Wayne Flye, Thalachallour Mohanakumar, Randy Bollinger, and Allan Kirk, and continues to impact scores of medical students, residents, and faculty, including the present authors (Figure 2). Through establishing the critical importance of HLA matching for successful kidney transplantation, cofounding the precursor to UNOS, making seminal discoveries in the potential of cross-species organ sharing, and pioneering canine cardiac transplant, his contributions continue to influence the field. His influence is well summarized in the words of the Chair of Surgery at Duke during Dr. Seigler's early career:

Many times I have reflected upon your numerous and significant contributions to the Department. You were very wise in obtaining fundamental training in immunology with Bernard Amos and have pursued the field brilliantly ever since. Your trainees in the laboratory have represented the Surgical Program extremely well and will be a great tribute to you in the future. In addition, you have been most successful in obtaining NIH and VA research support, which has meant much to our productivity and national image. Be assured that these are both recognized and greatly appreciated.

With my special thanks again to a wonderful friend and colleague and with very best wishes to you. David C. Sabiston, Jr., M.D.

## Data Availability

The original contributions presented in the study are included in the article, further inquiries can be directed to the corresponding author.
